# Effects of a 12-Week Functional Training Program on the Strength and Power of Chinese Adolescent Tennis Players

**DOI:** 10.3390/children10040635

**Published:** 2023-03-28

**Authors:** Wensheng Xiao, Xiaorong Bai, Soh Kim Geok, Dawei Yu, Yang Zhang

**Affiliations:** 1 School of Physical Education, Huzhou University, Huzhou 313000, China; 03218@zjhu.edu.cn (W.X.); 03219@zjhu.edu.cn (X.B.); 02674@zjhu.edu.cn (D.Y.); 2 Department of Sports Studies, Faculty of Educational Studies, Universiti Putra Malaysia, Serdang 43400, Malaysia; 3 Independent Researcher, Orlando, FL 34786, USA; yzhang68@crimson.ua.edu

**Keywords:** youth, resistance training, motor development, musculoskeletal adaptation, physical fitness

## Abstract

Background: Functional training is any type of training designed to improve a specific movement or activity for fitness or high performance sports. This study examined the effect of functional training on the strength and power of young tennis players. Methods: 40 male tennis players were assigned to the functional training group (n = 20; age, 16.7 ± 0.4 years) or the conventional training group (n = 20; age, 16.5 ± 0.6 years). The functional training group received three 60 min sessions per week for 12 weeks, while the conventional training group participated in three sessions per week of mono-strength exercise for 12 weeks. Strength and power were measured according to the International Tennis Federation protocol at baseline, 6 weeks after the intervention, and 12 weeks after the intervention. Results: Both forms of training increased (*p* < 0.05) push-ups, wall squat test, over medicine ball throw, and standing long jump after 6 weeks of training, and the effect improved further as the 12-week mark approached. Except for the wall squat test (left) at 6 weeks, functional training showed no advantage over conventional training. After an additional 6 weeks of training, all measures of strength and power were better (*p* < 0.05) in the functional training group. Conclusions: Improvements in strength and power could occur after as little as 6 weeks of functional training, and 12-week functional training could outperform conventional training in male adolescent tennis players.

## 1. Introduction

For many sports, strength and power are foundational qualities [1]. Modern tennis has evolved from a primarily technical sport dominated by sport-specific technical skills [2] to a dynamic, advanced sport characterized by stroke and serve speed, higher physical speed, and movement demands that are both strategic and explosive [3]. Therefore, modern tennis players must be physically fit to execute more complex shots and compete against increasingly formidable opponents. In addition, tennis requires a high level of muscle and joint strength for performance (ball velocity) and injury prevention (joint protection), while a sufficient range of motion in the major joints (e.g., rotator cuff muscles) is necessary for strokes and on-court movement [4]. Various training strategies have been developed to improve the strength and power of tennis players [5,6]. Similar to many other sports, there is no one-size-fits-all training method and no universally accepted method in tennis that can be recommended as the best way to improve the strength and power of tennis players.

Functional training is a trending method for enhancing the strength and power of athletes [7]. Functional training refers to the training of partial chains and connections in the human motion chain, which includes multi-dimensional motion trajectory acceleration, deceleration, and stability training activities that meet the characteristics of specific target actions [8]. The essence of functional training is goal-directed exercise, meaning it could consist of any exercise designed to improve a particular movement or activity [9]. In other words, if balance training is included in a functional training program, the training intervention aims to improve sport-specific balance performance. Unlike mono-strength exercise, functional training introduces a variety of movements and stimulates all of the major muscle groups in the body, hence its growing popularity in professional tennis training [10].

Functional training that emphasizes the development of strength and power differs from conventional resistance training, which focuses on increasing the strength and endurance of a specific muscle group [11]. Functional training, on the other hand, is designed to isolate and develop individual muscles through the use of body weight, stable positions, and fixed training equipment [12]. Moreover, conventional training often relies on high loads and duration to develop or enhance muscular performance [12]. However, tennis requires the simultaneous use of multiple muscle groups and joints across multiple axes [13], rendering conventional training inadequate for achieving the desired improvement in tennis players’ specific physical fitness.

Functional training has shown promise for enhancing the physical fitness of diverse populations. Green and colleagues conducted a cross-sectional study on male athletes with backgrounds in either strength, endurance, or high-intensity functional training [14]. It was observed that the functional training group had comparable power outputs and absolute strength to the strength-focused group. Interestingly, the functional training group exhibited fatigue resistance and mitochondrial capacity comparable to endurance training participants. Similarly, another study comparing the physiological profiles of trained athletes found that athletes who participated in high-intensity functional training attained aerobic levels comparable to those of endurance athletes and power and strength output comparable to those of power athletes [15]. It is possible for functional training to simultaneously improve multiple facets of physical fitness. These findings of Green et al. [14] and Adami et al. [15] have now been replicated in a randomized controlled trial. Compared to endurance training or strength training alone, functional training improved the counter-movement jump, 20 m sprint, 3-repetition maximum back squat, and yo-yo test in an untrained population after 6 weeks of training twice per week for 60–75 min per session [16].

Compared to adult populations, there are limited studies on functional training for children and adolescents. Previous studies focused on youth soccer players [17], martial artists [18], and handball players [19], whereas research on youth tennis players is scant [13]. For example, Zrhl and Demirci reported that 8 weeks of functional training improved the 10 m sprint, vertical leap, flexibility, hand gripping force, and T agility test in 10–12-year-old female tennis players [20]. However, the effects of functional training on other age groups of youth tennis players remain unknown. The effect of functional training on the strength and power performance of young tennis players, especially, is not yet backed up by research [12]. Furthermore, due to insufficient data, there is no consensus regarding how long a functional training intervention has to last to increase the strength and power of trained youth athletes. Given its potential in sports with a high demand for strength and power, it would be interesting to determine if young tennis players, who are in a crucial stage of physical fitness development, could reap the same benefits observed in other sports and adult populations.

Therefore, this study investigated the effect of 12-week functional training on the strength and power of young tennis players. The results are intended to assist coaches in optimizing their training methods, especially in developing countries where young players often lack access to advanced resistance training equipment.

## 2. Materials and Methods

### 2.1. Design

This research was approved by the Ethics Committee of Universiti Putra Malaysia (protocol number JKEUPM-2020-283) and parental consent was obtained. The participant’s legal guardian provided informed consent, and participants could withdraw from the study at any time.

**Figure 1:**
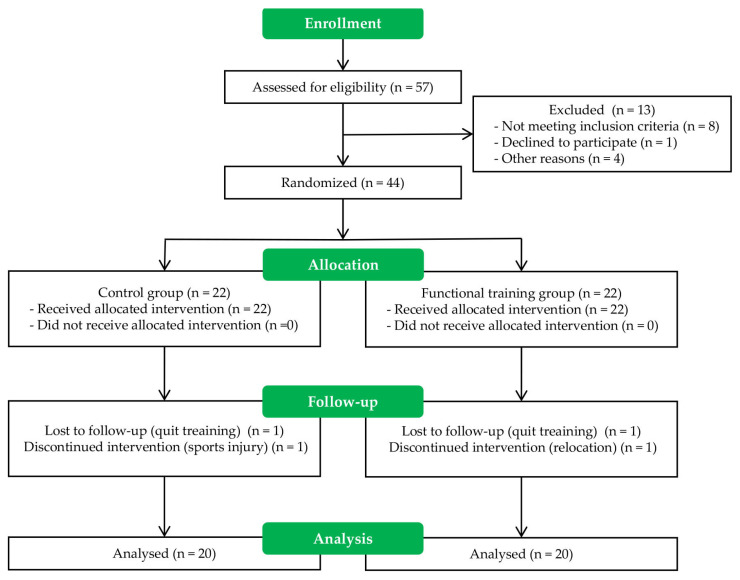
CONSORT flow diagram.

The study protocol is summarized in Figure 1. The design and reporting of the study followed the CONSORT statement [21] and a cluster-randomized approach. The sample size was determined using G*Power 3.1. The effect size was calculated using data from previous studies (effect size = 0.24). The sample size was calculated to be 30 (15 for each group) based on an alpha of 0.05 and a power of 0.80. To achieve the required statistical power in cluster randomized trials, the presumptive individual randomization sample size may be amplified by a design effect [22]. Taking into account the design effect and a potential dropout rate of 20% of the total sample size [23], the final sample size was determined to be 42.75, which was rounded to 44 tennis players.

Tennis players from the Youth Tennis Reserve Training Base in Jiaxing, Zhejiang province were recruited. Given that the majority of tennis players were male during the study period, only 14- to 18-year-old male tennis players were recruited for this study. Participants were removed from the final list if any of the following conditions were met: recent (i.e., less than one year) history of knee, elbow, or shoulder injury; history of rheumatoid disease or neurological damage for which treatment is ongoing [24]; prior experience with functional training in the 12 months before the present study; and players who are about to discontinue tennis training due to uncertain factors. Table 1 summarizes the participants’ demographics.

### 2.2. Intervention

During the study’s planning phase, seven experts familiar with tennis training assessed the content validity of the training intervention and outcome evaluation [25]. Specifically, the items content validity index (I-CVI) and modified Kappa coefficient factor were used to determine the content validity using a 4-point ordinal scale (1, ‘not relevant’; 2, ‘somewhat relevant’; 3, ‘quite relevant’; 4, ‘highly relevant’) [26]. Items with I-CVI values below 0.78 were adjusted or removed, and Kappa values above 0.75 indicate excellent content validity, between 0.40 and 0.75 indicate fair to good content validity, and below 0.40 indicate poor content validity. In addition, an open-ended questionnaire was provided to solicit expert feedback for micro-adjustments to the training intervention.

The functional training group and the conventional training group were trained every Monday, Wednesday, and Friday from 4–5 p.m. The functional training group received a program based on Santana’s Racket Sport Program [27], whereas the conventional training group followed the conventional training program. The functional training program details are presented in Table 2. Researchers collected the participants’ training logbooks weekly and encouraged them to adhere to the intervention. The definition of intervention adherence was attendance at 80% of prescribed sessions.

### 2.3. Evaluation

The evaluation of strength and power followed the International Tennis Federation (ITF) standard test protocol [28]. For this study, the ITF’s strength (push-ups and wall squat test) and power (over medicine ball throw and standing long jump) test batteries were utilized.

One week before the baseline evaluation, all participants and their coaches attended an orientation session. The participants were instructed to maintain their daily diet in the dining hall of the training base and to avoid alcoholic beverages. All participants were required to eat food in the dining hall 24 h before each evaluation. During this meeting, they were also given a familiarization trial with the ITF test batteries. To ensure optimal recovery, their coaches were instructed to refrain from assigning strenuous workouts 24 h before each evaluation.

All the data collection took place between 8 and 11 a.m. After completing a tennis-specific warm-up, each participant completed the tests. After the baseline evaluation, the test batteries were administered again after 6 and 12 weeks of the intervention. All of the testers majored in physical education, and two of them are certified ITF level-2 coaches.

### 2.4. Statistics

Data were analyzed using the IBM SPSS version 23. The homogeneity of two groups for demographics and outcome variables at baseline were examined using a two-tailed *t*-test. To determine the efficacy of the intervention, a generalized estimating equation analysis was conducted, followed by Bonferroni correction for pairwise comparison. Given that the average training experience of the sample was less than 5 years, the effect size (Cohen d) was interpreted as follows: trivial, 0.35; small, 0.35 to 0.80; moderate, 0.80 to 1.50; and large, >1.50 [29]. A p-value less than 0.05 was considered statistically significant.

## 3. Results

Based on the experts’ evaluation, both the relevancy (training intervention: I-CVI = 0.857–1.000, Kappa = 0.588–1.000; strength: I-CVI = 0.857–1.000, Kappa = 0.682–1.000; power: I-CVI = 0.857, Kappa = 0.682–0.741) and clarity (training intervention: I-CVI = 0.857–1.000, Kappa = 0.696–1.000; strength: I-CVI = 0.857–1.000, Kappa = 0.682–1.000; power: I-CVI = 0.857–1.000, Kappa = 0.588–0.741) met the acceptable thresholds, indicating that the content validity was satisfactory.

Table 3 summarizes the results of the ITF test batteries. Briefly, both forms of training improved strength and power, but the 12-week functional training showed greater promise among 14- to 18-year-old tennis players.

First, there was a significant time effect. At 6 weeks, both forms of training improved the push-ups, wall squat test, over medicine ball throw, and standing long jump. All measures of strength and power improved following an additional 6-week training. Overall, after 12 weeks, both forms of training had a large training effect on the push-ups and wall squat test (left); functional training had a large training effect on the wall squat test (right) and standing long jump; conventional training had a moderate training effect on the wall squat test (right) and standing long jump, and both forms of training had a small training effect on the over medicine ball throw.

Second, there was a significant group effect. At 6 weeks, only the wall squat test (left) was different between the two forms of training. At 12 weeks, the functional training group outperformed the conventional training group on all measures of strength and power. Compared to conventional training, 12-week functional training had a large training effect on the wall squat test (left), a moderate training effect on the push-ups and standing long jump, and a small training effect on the wall squat test (right) and over medicine ball throw.

## 4. Discussion

This study demonstrates that functional training for as little as 6 weeks can improve the strength and power of adolescent male tennis players. These results are expected and consistent with the population studied. Children’s strength can increase by 30% to 50% after just 8 to 12 weeks of a well-designed strength training program [30]. Nonetheless, when considering the use of functional training to replace conventional training on strength and power development, our results suggest that training may require 12 weeks to yield superior results. These findings are essential for guiding the coaching of adolescent tennis players, whose motor development is crucial at this age.

Existing evidence suggests that functional training can enhance strength and power in a variety of sports. Oliver and Brezzo [31] found that the strength (single-leg squat) of female volleyball players increased following 13 weeks of functional training. Alonso-Fernández et al. [19] reported that 8 weeks of functional training improved the repeated sprint ability of female handball players. Park [32] found that 6 weeks of functional training increased the back strength of elite taekwondo athletes. Fernandez-Fernandez et al. [33] found that 8 weeks of neuromuscular warm-up before and after tennis-specific training increased muscular strength and power as measured by counter-movement jump, medicine ball throw, and shoulder strength. Collectively, this study not only adds additional evidence to the growing body of literature on functional training for youth but also demonstrates that a 6-week functional training program is effective in increasing both strength and power in trained male adolescent tennis players.

The notable point seemed to be that 6 weeks of functional training cannot lead to higher strength and power than conventional training. At the 6-week mark, the functional training group only performed better on the wall squat test (left). This difference could be because the dominant hand of the participants is the right hand (forehand) and their support foot is the left foot. In this sample, all athletes hold the tennis racket with their right hand (forehand shot). In professional tennis, the forehand shot is the most used and essential for offensive scoring [34]. Accordingly, when the tennis ball is struck, the typical supporting foot is left [35]. In addition, the left foot is the foot that lands after striking the tennis ball, which can help strengthen the left lower body [36]. Therefore, this condition may account for the significant difference observed after 6 weeks of functional training, indicating that functional training may be an efficient method for enhancing underdeveloped strength. This finding, however, does not support the notion that 6 weeks of functional training improved other strength and power components more than conventional training.

According to previous research, as little as 4 weeks of functional training is sufficient to produce greater gains in strength and power than conventional training. For example, Yildiz et al. [13] reported that a 4-week functional training improved the functional movement screen in comparison to conventional training. Likewise, Tomljanovi et al. [37] found that 5 weeks of functional training improved the strength (standing overarm and lying medicine ball throw) of moderately trained male athletes (aged from 22 to 25 years). This inconsistency may be attributable to a variety of confounding variables, including training experience, the volume of functional training, and evaluation objectives. It is important to note that in the aforementioned studies, the movement patterns in the functional training were similar to their sports-specific tests, which could help athletes adapt to training. This might explain why 1 month of functional training could have a noticeable effect.

In this study, an additional 6 weeks of functional training outperformed in all measures of strength and power. This is consistent with the findings obtained by Yildiz et al. [13] from a group of school-aged tennis players. Although their functional training group demonstrated significant improvement on the functional movement screen at the 4-week mark compared to the conventional training group, their functional training showed the most promise after an additional 4 weeks of training. Specifically, functional training outperformed conventional training in terms of flexibility, counter-movement jump, T-test agility, dynamic balance (both left and right), static balance, and the functional movement screen over an 8-week duration. Our results not only corroborate these preliminary findings but also demonstrate that 12-week functional training could provide larger advantages than conventional training for adolescents who are engaging in systematic tennis training. In terms of effect size, functional training demonstrates remarkable advantages in tennis-specific lower-limb strength and power, which could be particularly beneficial for enhancing on-court performance and reducing injury risks [38]. Particularly, the wall squat test (left) revealed an exceptionally large effect size, indicating that functional training may be especially useful in developing the supporting foot strength of young tennis players.

The difference in subsequent gains in strength and power between functional and conventional training after 6 weeks may be attributable to the plateau effect in sports training [39]. Resistance training induces changes in motor unit recruitment during voluntary contractions. The onset of training adaptation is associated with corticospinal signaling neural adaptations [40], and significant changes in motor unit behavior, such as a concurrent increase in discharge rate and decrease in the recruitment-threshold force of motor units, could occur as early as 3 weeks [41]. Nonetheless, such adaptation often moderates [42] or even normalizes over time [43], resulting in a plateau phase following additional training exposure. For example, rapid changes in motor unit discharge rate occurred in the early 3-week phase of strength training compared to later 3-week phases in untrained young adults [41]. Current literature suggests that simple overload in training duration becomes less effective after 3–6 weeks of compensatory adaptations and that introducing controlled overload in training intensity and/or variation is recommended for eliciting additional performance gains. This aligns with the present findings. In the first half of the training program, both strength and power increased substantially. Nevertheless, conventional tennis training does not introduce variation after the onset of adaptation, whereas functional training is exceptional in terms of training variation and intensity (see also Table 2) throughout the second half of the training program, making it superior to conventional training after the entire training program. This study not only provides additional empirical data regarding the development curve of strength and power in trained adolescents, but it also sheds new light on the design of a mixed resistance training program for adolescent tennis players.

While the focus of this study was on the utility of functional training, it is also important to consider the logistics of this type of training. In China, and likely in other developing countries, training facilities for young athletes beginning systematic training may be limited and insufficient. Functional training has the advantage of using inexpensive resistance devices (e.g., dumbbells, medicine balls) in daily training [27], which could be convenient yet highly effective when designing resistance training programs for children and adolescents residing in developing countries.

This study has a few limitations. Only the strength and power components of physical fitness were investigated in this study. Future research should look into the effect of functional training on other sport-specific components of physical fitness. Although the caloric intake of the two groups should be comparable, we did not control for energetic beverages such as coffee consumption during the study period. It should be noted that traditionally, the Chinese population does not consume coffee on a regular basis. However, as coffee popularity increases among the young Chinese generation, it is possible that the lack of caffeine consumption control prior to the evaluation may confound the results. In addition, there was no traditional control group, as it would be unethical to discontinue regular training programs. Consequently, the size of the final effect may be inflated due to the normal physical development of adolescents over 3 months. Notwithstanding only male tennis players being recruited for this study, we are cautiously optimistic about the generalization of its efficacy to female tennis players, though a confirmation study is still needed. Finally, although the present sample population is deemed to be beyond the age-specific maturity status in tennis, future research may consider reporting the peak-height velocity for players younger than 16 years of age [44]. In practice, peak-height velocity would enable coaches and team sports scientists to better select and intervene in the training of youth athletes.

In conclusion, this study provides strong evidence regarding the benefits of a 12-week functional training program for youth tennis players. To maximize the continuous development of strength and power, coaches for this age group could incorporate functional training such as our protocol into their routine training regimens, which could be particularly beneficial for young athletes from developing countries.

## Figures and Tables

**Table 1:** Participants’ demographics.

Variables	CT	FT	p
	M (SD)	M (SD)	
Age (year)	16.5 (0.6)	16.7 (0.4)	0.360
Height (cm)	176.4 (2.4)	176.2 (2.6)	0.836
Weight (kg)	71.8 (3.2)	71.6 (3.0)	0.830
Training experience (month)	58.0 (4.2)	57.9 (4.4)	0.971

Note: CT: conventional training group; FT: functional training group.

**Table 2:** A detailed description of the 12-week functional training.

**Type of Exercise**	**Week 1**	**Week 2**	**Week 3**	**Week 4**
MB wood chop Side T plank BP compound row	2 sets × 10 reps	3 sets × 10 reps	3 sets × 15 reps	4 sets × 10 to 15 reps
MB ABC squat BP staggered-stance fly BP staggered-stance CLA row	2 sets × 10 reps	3 sets × 10 reps	3 sets × 15 reps	2 sets × 10 to 15 reps
Rope circles (clockwise and counterclockwise) Vibration blade throw (per side)	2 sets × 10 to 15 s 2 sets × 10 s			
Frequency, times, intensity, and rest	3 times/weeks, 60 min, 60–75% 1RM, 30–60 s			
**Type of Exercise**	**Week 5**	**Week 6**	**Week 7**	**Week 8**
BP low-to-high chop DB single-arm diagonal fly rotation BP staggered-stance CLA compound row	1 set × 6 reps	2 sets × 6 reps	3 sets × 4 reps	4 sets × 4 reps
DB or KB lateral reaching lunge T push-up DB or KB staggered-stance bent-over single-arm row	1 set × 6 reps	2 sets × 6 reps	3 sets × 4 reps	4 sets × 4 reps
X-up SB rollout Rope circles (clockwise and counterclockwise) Vibration blade throw (per side)	2 sets × 10 reps 2 sets × 10 reps 2 sets × 20 s 2 sets × 15 s			
Frequency, times, intensity, and rest	3 times/weeks, 60 min, 75–90% 1RM, 30–60 s			
**Type of Exercise**	**Week 9**	**Week 10**	**Week 11**	**Week 12**
DB or KB lateral reaching lunge Skater	2 sets × 5 + 5 reps	3 sets × 5 + 5 reps	3 or 4 sets × 5 + 5 reps	4 sets × 5 + 5 reps
BP low-to-high chop MB rotational throw: perpendicular	2 sets × 5 + 5 reps	3 sets × 5 + 5 reps	3 or 4 sets × 5 + 5 reps	4 sets × 5 + 5 reps
Biplex 3 BP high-to-low chop MB overhead side-to-side slam	2 sets × 5 + 5 reps	3 sets × 5 + 5 reps	3 or 4 sets × 5 + 5 reps	4 sets × 5 + 5 reps
BP swim MB overhead slam	2 sets × 5 + 5 reps	3 sets × 5 + 5 reps	3 or 4 sets × 5 + 5 reps	4 sets × 5 + 5 Reps
Single-leg CLA anterior reach (per leg) Rope circles (clockwise and counterclockwise) Vibration blade throw (per side)	3 sets × 10 reps 3 sets × 10 to 15 s 3 sets × 10 s			
Frequency, times, intensity, and rest	3 times/weeks, 60 min, 95–100% 1RM, 1–2 min			

Note: 1RM: one repetition maximum; 5 + 5: 60 s of rest between the first and second exercise; BP: bands or pulleys; CLA: contralateral arm; DB: dumbbell; KB: kettlebells; MB: medicine balls; SB: stability balls.

**Table 3:** Tennis-specific performance following 12-week training.

Test Battery		Time	Measurement		Between-Group		Within-Group d (T0 vs. T12)	
			CT	FT	p	d	CT	FT
Strength	PU (#)	T0	38.9 (1.7)	38.9 (1.7)	1.000	<0.001	1.62	3.44
		T6	41.0 (3.6) *	41.2 (2.8) *	0.802	0.08		
		T12	44.8 (4.9) †	48.3 (3.5) †	0.008	0.82		
	WSTL (sec)	T0	36.0 (2.5)	37.2 (2.4)	0.136	0.46	1.93	4.40
		T6	38.5 (2.4) *	43.3 (2.8) *	<0.001	1.85		
		T12	41.2 (2.8) †	47.8 (2.4) †	<0.001	2.51		
	WSTR (sec)	T0	57.5 (2.9)	57.1 (3.6)	0.683	0.13	0.98	1.59
		T6	59.4 (2.9) *	60.1 (3.5) *	0.444	0.24		
		T12	60.3 (2.9) †	62.7 (3.5) †	0.017	0.73		
Power	OMBT (m)	T0	8.9 (1.4)	9.4 (1.6)	0.249	0.36	0.45	0.65
		T6	9.2 (1.4) *	9.8 (1.5) *	0.202	0.39		
		T12	9.5 (1.3) †	10.4 (1.4) †	0.033	0.66		
	SLJ (m)	T0	2.52 (0.11)	2.54 (0.12)	0.469	0.22	0.92	1.51
		T6	2.56 (0.11) *	2.61 (0.10) *	0.132	0.46		
		T12	2.62 (0.10) †	2.72 (0.12) †	0.002	0.95		

Note: CT, conventional training; FT, functional training; T0, pre-intervention test; T6, 6-week post-intervention test; T12, 12-week post-intervention test; PU, push-ups; WSTL, wall squat test (left); WSTR, wall squat test (right); OMBT, over medicine ball test; SLJ, standing long jump. * T0 vs. T6, *p* < 0.05; † T6 vs. T12, *p* < 0.05.

## Data Availability

The data that support the findings of this study are available from the first author, W.X., upon reasonable request.
